# Dataset for effect of elevation on the insect herbivory of Mongolian oaks in the high mountains of southern South Korea

**DOI:** 10.1016/j.dib.2019.104799

**Published:** 2019-11-13

**Authors:** Sei-Woong Choi, Jae-Cheon Sohn, Nang-Hee Kim

**Affiliations:** a Department of Environmental Education, Mokpo National University, Muan, Jeonnam, 58554, Republic of Korea; b Department of Science Education, Gongju National University of Education, Gongju, Chungnam, 32553, Republic of Korea; c Division of Industrial Insect, National Institute of Agricultural Sciences, Rural Development Administration, Wanju, Jeonbuk, 55365, Republic of Korea

**Keywords:** Insect-feeding damage types, Herbivory, Mongolian oak

## Abstract

The data presented in this article are related to the research article “Effect of elevation on the insect herbivory of Mongolian oaks in the high mountains of southern South Korea” (Sohn et al., 2019). We collected oak leaves occurring in two mountains: Jirisan Mountain on the mainland of Korea (12 September 2015) and Hallasan Mountain on Jejudo Island (21 September 2015). From three randomly-chosen trees, we sampled six branches with breast height with all leaves attached. Feeding traces associated with feeding activities of insects and mites on the leaves were recorded. The underlying data of that research article are presented here: Feeding damage type per surveyed leaf at four research sites of southern South Korea; the geographic location of the study sites on each mountain and the abbreviation of the feeding types and guilds.

**Table:** Specifications Table

Subject	Ecology
Specific subject area	Herbivory, Insect ecology, Community ecology
Type of data	Excel file
How data were acquired	We collected oak leaves occurring in two high mountains: Jirisan National Park on the mainland of Korea (12 September 2015) and Hallasan National Park on Jejudo Island (21 September 2015). From three randomly-chosen trees, we sampled six branches with breast height with all leaves attached.Leaves of all ages were detached from the collected branches, flattened using a standard plant press (BioQuip Co., USA). Feeding traces associated with feeding activities of insects and mites on the leaves were counted under a stereoscope (Leica EZ4, Leica Co.).
Data format	Raw and Filtered
Parameters for data collection	Oak leaf samples were collected on 12 September 2015 on Hallasan and on 21 September 2015 on Jirisan Mountain. Six branches at breast height (about 1.2 m above the ground) with all leaves attached were sampled from three randomly-chosen trees. Feeding traces associated with feeding activities of insects and mites on the leaves were counted under a stereoscope (Leica EZ4, Leica Co.).
Description of data collection	Types of insect-feeding damage (hole feeding, skeletonization, marginal feeding, surface feeding, piercing/sucking, mining and galling) were determined using their morphological characteristics, positions on the leaves, and interactions with the foliar venation. Three feeding guilds (external feeding, internal feeding, and piercing/sucking) were also determined.
Data source location	Sample was collected from two different elevations of southern South Korea. Mount. Jirisan National Park: Seongsamjae (SSJ) 35˚18′20.8″N 127˚30′44.6″E, Sangseonam (SSA) 35˚17′31.5″N 127˚29′39.4″E; Mount. Hallasan National Park: Sajebi Hill (SJB) 33˚22′32.2″N 126˚29′58.8″E, Lower Yongsil (LYS) 33˚20′54.8″N 126˚29′47.6″E.
Data accessibility	Data are available in this article. Mendeley data (https://doi.org/10.17632/ybt55vwzs7.1)
Related research article	Sohn, J.-C., N.-H. Kim, S.-W. Choi, Effect of elevation on the insect herbivory of Mongolian oaks in the high mountains of southern South Korea. Journal of Asia-Pacific Entomology 22 (2019), 957–962 [1]. Sohn, J.-C. N.-H. Kim, S.-W. Choi, Morphological and functional diversity of foliar damage on Quercus mongolica Fisch. ex Ledeb. (Fagaceae) by herbivorous insects and pathogenic fungi. Journal of Asia-Pacific Biodiversity 10 (2017) 489–508. https://doi.org/10.1016/j.japb.2017.08.001 [2]

**Table:** 

**Value of the Data** • This data article focuses on the different insect feeding damage types on a Mongolian oak tree (*Quercus mongolica*) at two high elevations • The data are useful to compare the feeding types and amount of damages by insects and other arthropods on oak trees that are about 600 species in the Northern Hemisphere. • Data provided in this article can be used by ecologists to plot correlations between physical and chemical properties of plants and feeding behaviors of herbivorous arthropods and to compare feeding damages along elevational gradient. • Ecologists can use this information to better understand insect herbivory pattern of different feeding guilds (e.g. chewer, galler, miner, and piercing and sucker) to broad-leaves tree species in temperate region.

## 1 Data

**Fig. 1:**
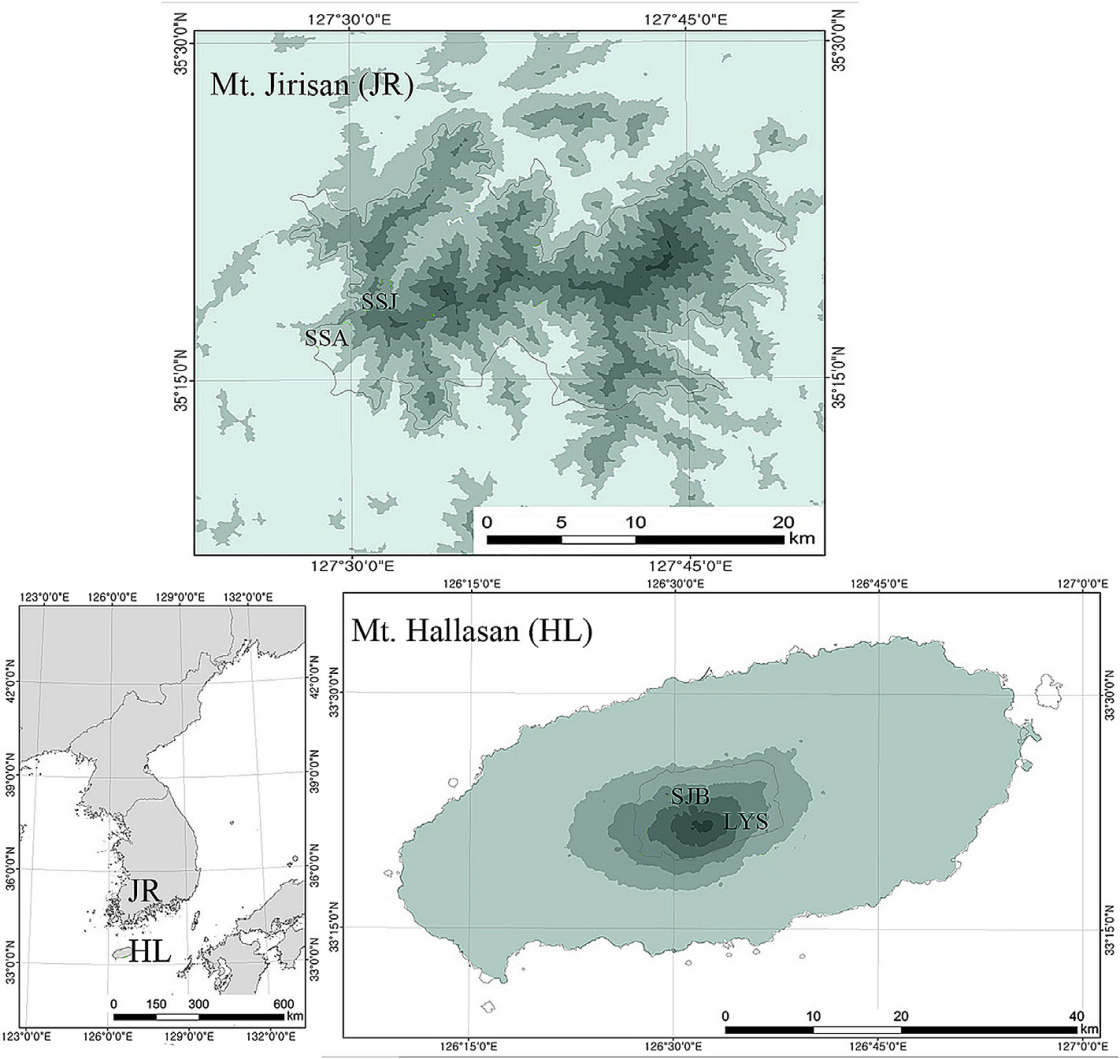
Map of the study sites in Jirisan Mountain (JR) and Hallasan Mountain (HL), South Korea.

This Data in Brief article provides one figure and the raw data describing the feeding damage by insects and other arthropods on Mongolian oak trees from four high elevation sites of southern South Korea. The dataset contains 4452 feeding damages on 572 oak tree leaves: LYS (131 leaves) and SJB (199 leaves) from Hallasan (HL), and SSA (106 leaves) and SSJ (136 leaves) from Jirisan (JR) mountain (Fig. 1). Each feeding type is compatible to the feeding type in Sohn et al. (2017) [2] and these 90 feeding types are assigned to nine feeding damage types, FG (fungal), GA (galler), HF (hole feeding), IS (*incertae sedis*), MF (marginal feeding), MI (mining), PS (piercing and sucking), SF (surface feeding), and SK (skeletonization). These feeding damage types are classified into five feeding guilds, external feeding (HF, MF, SF, SK), internal feeding (GA, MI), piercing/sucking (PS), fungal (FG) and *incertae sedis* (IS). These data are discussed in the reference article [1].

## 2 Experimental design, materials, and methods

We examined the insect herbivory on Mongolian oak trees (*Quercus mongolica*) occurring in two high mountains: Jirisan National Park on the mainland of Korea and Hallasan National Park on Jejudo Island (Fig. 1).

Oak leaves were collected on 12 September 2015 on Hallasan mountain and on 21 September 2015 on Jirisan mountain. Only branches at breast height (about 1.2 m above the ground) were considered for checking and six branches with all leaves attached were sampled from three randomly-chosen trees. To minimize possible edge effects, trees at least 10 m away from trails were surveyed. Leaves of all ages were detached from the collected branches, flattened using a standard plant press (BioQuip Co., USA), and stored in a freezer.

Feeding traces associated with feeding activities of insects and mites on the leaves were counted under a stereoscope (Leica EZ4, Leica Co.) and photographed as necessary, using a digital camera (Nikon D30, Nikon Co.) attached to a stereoscope (Leica L2, Leica Co.).

Types of insect-feeding damage (hereafter abbreviated as DT [ = damage type]) were determined using their morphological characteristics, positions on the leaves, and interactions with the foliar venation. The feeding traces were categorized referring to Labandeira et al. (2007) [3], with some modification (Sohn et al., 2017 [2]): hole feeding (HF), skeletonization (SK), marginal feeding (MF), surface feeding (SF), piercing/sucking (PS), mining (MI), and galling (GA). Three feeding guilds were assigned: external feeding (SF, MF, HF, SK), internal feeding (MI, GA), and piercing/sucking (PS). The feeding types that couldn't be assigned to any of the above categories were treated as *incertae sedis* (IS).
